# Omega-3 fatty acid and menaquinone-7 combination are helpful for aortic calcification prevention, reducing osteoclast area of bone and Fox0 expression of muscle in uremic rats

**DOI:** 10.1080/0886022X.2022.2142140

**Published:** 2023-01-12

**Authors:** Su Mi Lee, Eu Gene Jeong, Yu In Jeong, Seo Hee Rha, Seong Eun Kim, Won Suk An

**Affiliations:** a Department of Internal Medicine, College of Medicine, Dong-A University, Busan, Republic of Korea; b Department of Pathology, College of Medicine, Dong-A University, Busan, Republic of Korea

**Keywords:** Menaquinone, omega-3 fatty acid, osteopenia, sarcopenia, uremia

## Abstract

**Background:**

Osteopenia, sarcopenia, and vascular calcification (VC) are prevalent in patients with chronic kidney disease and often coexist. In the absence of proven therapies, it is necessary to develop therapeutic or preventive nutrients supplementation for osteopenia, sarcopenia, and VC. The present study investigated the effect of omega-3 fatty acid (FA) and menaquinone-7 (MK-7) on osteopenia, sarcopenia, and VC in adenine and low-protein diet-induced uremic rats.

**Methods:**

Thirty-two male Sprague-Dawley rats were fed diets containing 0.75% adenine and 2.5% protein for three weeks. Rats were randomly divided into four groups that were fed diets containing 2.5% protein for four weeks: adenine control (0.9% saline), omega-3 FA (300 mg/kg/day), MK-7 (50 µg/kg/day), and omega-3 FA/MK-7. Von Kossa staining for aortic calcification assessment was performed. Osteoclast surface/bone surface ratio (OcS/BS) of bone and muscle fiber were analyzed using hematoxylin and eosin staining. Osteoprotegerin (OPG) immunohistochemical staining was done in the aorta and bone. Molecules related with sarcopenia were analyzed using western blotting.

**Results:**

Compared to the normal control, OcS/BS and aortic calcification, and OPG staining in the aorta and bone were significantly increased in the adenine controls. OPG staining and aortic calcification progressed the least in the group supplemented with both omega-3 FA/MK-7. In the adenine controls, the regular arrangement of muscle fiber was severely disrupted, and inflammatory cell infiltration was more prominent. These findings were reduced after combined supplementation with omega-3 FA/MK-7. Furthermore, decreased mammalian target of rapamycin and increased Forkhead box protein 1 expression was significantly restored by combined supplementation.

**Conclusions:**

Combined nutrients supplementation with omega-3 FA and MK-7 may be helpful for aortic VC prevention, reducing osteoclast activation and improving sarcopenia-related molecules in adenine and low-protein diet induced uremic rats.

## Introduction

In patients with chronic kidney disease (CKD), osteopenia, sarcopenia, and vascular calcification (VC) are prevalent and often coexist [1,2]. In the uremic state, high serum calcium and phosphate concentrations, inflammation, and oxidative stress are known to be the major causes of VC [3]. Under these conditions, vascular endothelial cells transform into osteoblast-like cells and VC proceeds. VC is also caused by an imbalance between the mechanisms of calcium deposition in osteoblast-like endothelial cells and osteoblasts in the bone [4,5]. The incidence of fractures in patients undergoing dialysis is twice as high as that of the general population [6]. Sarcopenia commonly occurs in the elderly and in patients with CKD, and the resulting disability increases social costs and degrades quality of life. Several conditions associated with CKD, including low nutrient intake, physical inactivity, and metabolic acidosis, can enhance muscle wasting through increased protein degradation and decreased protein synthesis [7].

In the absence of proven therapies, it is necessary to develop therapeutic agents or preventive medicines for osteopenia, sarcopenia, and VC for advanced CKD. Omega-3 fatty acids (FAs), including eicosapentaenoic acid, are known to have beneficial effects on cardiovascular disease (CVD) and mortality [8,9]. Notably, omega-3 FAs attenuate arterial medial calcification, improve vascular endothelial function, reduce inflammatory response, and inhibit thrombogenesis [10]. Vitamin K is a cofactor for activation of matrix-Gla-protein (MGP), a potent inhibitor of arterial calcification [11]. Therefore, omega-3 FA and vitamin K supplementation could be a viable supplementation option to reduce VC. Because of the close association between osteopenia, sarcopenia, and VC, effective drugs may simultaneously affect on bone, muscle, and vessel. However, the effect of omega-3 FA and vitamin K supplementation on osteopenia and sarcopenia is currently unclear.

This study investigated the effect of omega-3 FA and vitamin K_2_ (menaquinone-7, MK-7) on VC, osteopenia and sarcopenia in adenine and low-protein diet-induced uremic rats.

## Materials and methods

### Animals and experimental design

Male Sprague-Dawley rats, initially weighing 330-350 g, were housed in cages in a temperature- and light-controlled room, with free access to water. All procedures were approved by the Dong-A University Institutional Animal Care Committee (DIACUC-approval-16-9).

Thirty-two adenine-induced uremic rats were fed diets containing 0.75% adenine and 2.5% protein (Envigo Teklad, Madson, WI, USA) for three weeks. After three weeks, rats were randomly divided into four groups, which were supplemented and fed diets containing 2.5% protein for four weeks. These groups included an adenine control group (0.9% saline by gastric gavage), an omega-3 FA-supplemented group (300 mg/kg/day by gastric gavage), an MK-7-supplemented group (50 µg/kg/day by gastric gavage), and a combined omega-3 FA and MK-7-supplemented group [12,13]. Six normal control rats were fed diets containing 2.5% protein for seven weeks. All animals were fed evenly and had access to water *ad libitum*. Rats were anesthetized with diethyl ether, and blood samples were obtained from the heart. Serum creatinine, blood urea nitrogen (BUN), and phosphorus levels were measured by an automatic analyzer (Roche, Germany).

### Histopathologic examination

After sacrifice, the muscle, bone, and aortic tissues were excised. After washing with heparinized saline, the soleus muscle tissues were fixed in 10% buffered formalin. The tibial bones were rinsed in running tap water for 24 h and incubated with a 10% EDTA (pH 7.4) solution for one month at 4 °C, for decalcification. The EDTA solution was changed daily. The tissues were embedded in paraffin and 4 μm sections were processed for staining with hematoxylin and eosin (H&E). Von Kossa staining with a calcium stain kit (Scytek Laboratories, 2513 BH Hague, Netherlands) that contained 5% silver nitrate, 5% sodium thiosulfate, and nuclear fast red was performed to detect aortic wall calcification. The morphology of the tissues was evaluated by a Pannoramic MIDI (3DHISTECH Ltd, Budapest, Hungary), and the calcification area was measured. The osteoclast surface-to-bone surface ratio (OcS/BS %) was determined using a quantitative stereological method for histology known as the Weibel technique [14].

### Immunohistochemical staining

The aorta and bone sections from the rats were incubated with 10 mM sodium citrate solution (pH 6.0) in an 80 °C overnight for antigen retrieval. Then, 3% H_2_O_2_ buffer and 5% normal goat serum were used to block endogenous peroxidase activity. The tissue sections were incubated with an anti-osteoprotegerin (OPG) antibody overnight at 4 °C and then with a matching secondary antibody for 1 h at 37 °C. The negative control slides were using buffer solution instead of the primary antibody. For the sections staining expression, 3,3-diaminobenzidine + H_2_O_2_ substrate and hematoxylin were used. The results were viewed using a Pannoramic MIDI.

### Western blot analysis

After muscle tissues were homogenized with PRO-PREP solution (Intron biotechnology, Seongnam, Korea) and incubated at 4 °C for 30 min. Protein lysates were centrifuged at 14,000 rpm for 20 min at 4 °C. Equal microgram of protein were loaded onto 7.5–15% sodium dodecyl sulfate polyacrylamide gel electrophoresis. Then, the proteins were transferred to a nitrocellulose membrane (Amersham Pharmacia Biotech, Piscataway, NJ, USA) and incubated with each antibody.

Antibodies against myostatin and insulin-like growth factor-1 (IGF-1) were purchased from Abcam (Cambridge, MA, USA). Antibodies against myogenin were obtained from DSHB (Iowa City, Iowa, USA). Antibodies against MyoD, interleukin-6 (IL-6), nuclear factor kappa B (NF-kB), inhibitor of kappa B (IkB), muscle atrophy F-box protein (MAFbx), muscle-specific ring finger protein1 (MuRF1), and glyceraldehyde-3-phosphate dehydrogenase (GAPDH) were obtained from Santa Cruz Biotechnology (Santa Cruz, CA, USA). Rabbit polyclonal antibodies against mammalian target of rapamycin (mTOR), phosphorylated serine/threonine‐specific protein kinase, phosphorylated phosphatidylinositol 3-kinase (p-PI3K), forkhead box protein 1 (Fox01), and forkhead box protein 3a (Fox03a) were purchased from Cell Signaling Technology (Beverly, MA, USA). The membranes were then incubated with a horseradish peroxidase-conjugated secondary antibody. For immunostaining with antibodies, Super Signal West Pico (Thermo Scientific, Hudson, NH, USA) was used to enhance the chemiluminescence substrate and detected with a LAS-3000 Plus imager (Fuji Photo Film, Tokyo, Japan). ImageJ (version 1.48q) was used to quantify and normalize the samples to the GAPDH control band.

### Statistical analysis

All quantitative data are presented as mean ± SD and analyzed by Mann–Whitney U-test or Kruskal–Wallis test using the SPSS 18.0 software (IBM Corp., Armonk, NY, USA). Differences with *p* < 0.05 were considered statistically significant.

## Results

### Laboratory data

Body weight at the end of the experiments was significantly reduced in the adenine control group compared to those in the normal control group (Table 1). In addition, the loss of body weight in the adenine control group was the highest among the five groups.

**Table 1.** Characteristics of the rats in each experimental group.

	Normal control	Adenine control	Adenine rats with omega-3 FA	Adenine rats with MK7	Adenine rats with omega-3 FA and MK7	*p*-value
Body Weight loss (g)	1 ± 9	104 ± 24*	89 ± 18*	92 ± 18*	85 ± 16*	<0.001
BUN (mg/dL)	8.4 ± 2.2	204.9 ± 87.7*	54.0 ± 57.3	58.07 ± 33.10	41.68 ± 17.92^a^	0.01
Creatinine (mg/dL)	0.5 ± 0.1	5.5 ± 0.9*	3.9 ± 0.4*	3.73 ± 0.22*	3.53 ± 0.46*	<0.001
Calcium (mg/dL)	11.3 ± 0.3	8.7 ± 0.4	8.9 ± 0.7	9.12 ± 0.79	8.85 ± 0.86	0.082
Phosphorus (mg/dL)	9.7 ± 0.5	32.4 ± 6.2*	22.5 ± 1.6	21.47 ± 1.75	20.97 ± 2.09	0.002

Data are expressed as means ± SD.

*p < 0.05 (mean values are significantly different from normal control).

^a^*p* < 0.05 (mean values are significantly different from adenine control).

The levels of BUN, serum creatinine, and phosphorus were significantly higher in the adenine control group than in the normal control group. In particular, BUN levels were significantly lower in the omega-3 FA/MK-7 supplementation group than in the adenine control group. However, the levels of BUN, serum creatinine, and phosphorus did not differ between omega-3 FA or MK-7 single supplementation groups compared to the adenine control group. Serum calcium levels also did not significantly differ among the five groups (Table 1).

### Aortic calcification and immunohistochemical staining of OPG in aorta

**Figure 1.**
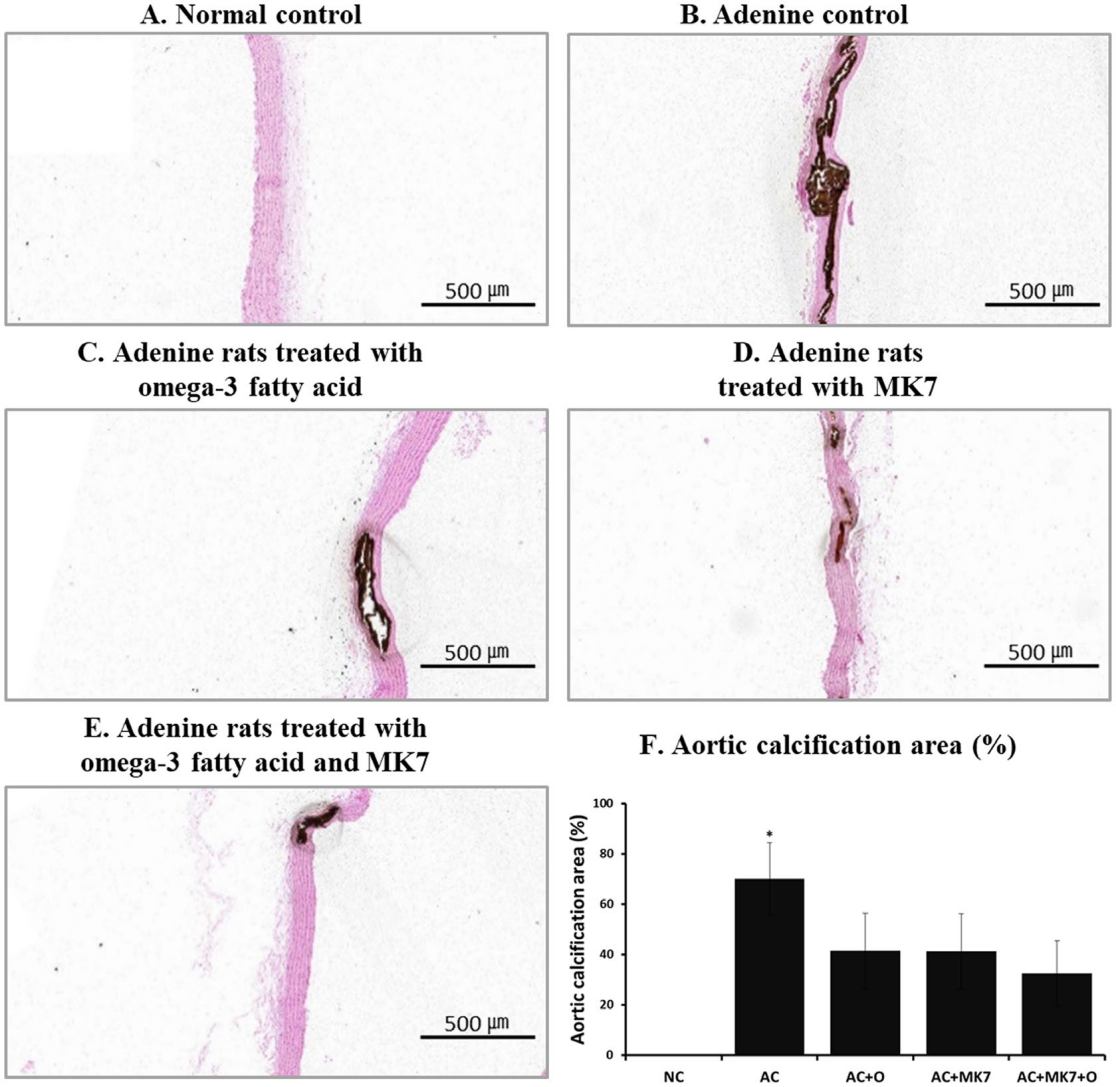
Aortic calcification area using von Kossa stain. Aortic calcification areas were prominent in adenine control group treated with 0.9% saline (1 mL/kg/day by gastric lavage) and was the least in supplemented group with combination of omega-3 FA (300 mg/kg/day by gastric lavage) and MK-7 (50 µg/kg/day by gastric gavage).

After four weeks of supplementation, aortic calcification on von Kossa stain analysis was progressed in the adenine control group compared to normal control group (Figure 1). Compared to adenine control group, less progressed aortic calcification was found in the omega-3 FA or MK-7 supplementation group. Aortic calcification was the least advanced in the group supplemented with a combination of omega-3 FA and MK-7, compared to the individual omega-3 FA or MK-7 supplementation group.

**Figure 2.**
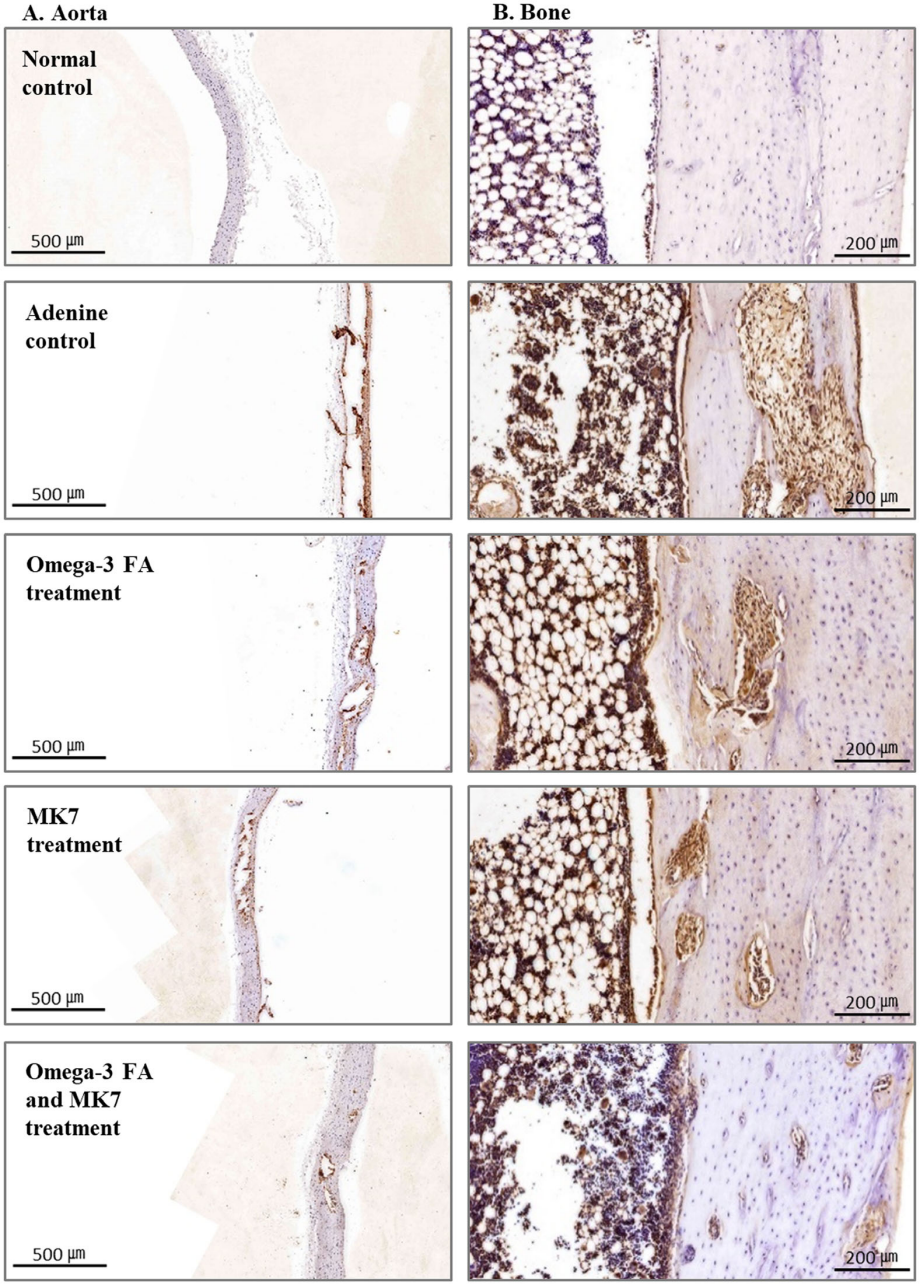
Immunohistochemical staining of osteoprotegerin (OPG) in the aorta (A) and bone (B). In the aorta and bone of the adenine control group, OPG stain was prominent compared to the expression in the normal control group, and this staining was decreased in omega-3 FA and MK-7 supplementation group.

Immunohistochemical (IHC) staining of OPG in the aorta is shown in Figure 2(A). IHC staining of OPG was prominent in the VC area of aorta. In the aorta of the adenine control group, OPG stain was prominent compared to the expression in the normal control group, and this staining was not prominent in omega-3 FA and MK-7 supplementation group.

### Immunohistochemical staining of OPG in bone and osteoclast surface-to-bone surface ratio

IHC staining of OPG in the tibia bone is shown in Figure 2(B). IHC staining of OPG was prominent in the osteoclast area. In the adenine control group, OPG expression was prominent compared to the normal control group, and this expression was not prominent in the omega-3 FA and MK-7 combination supplementation group.

**Figure 3.**
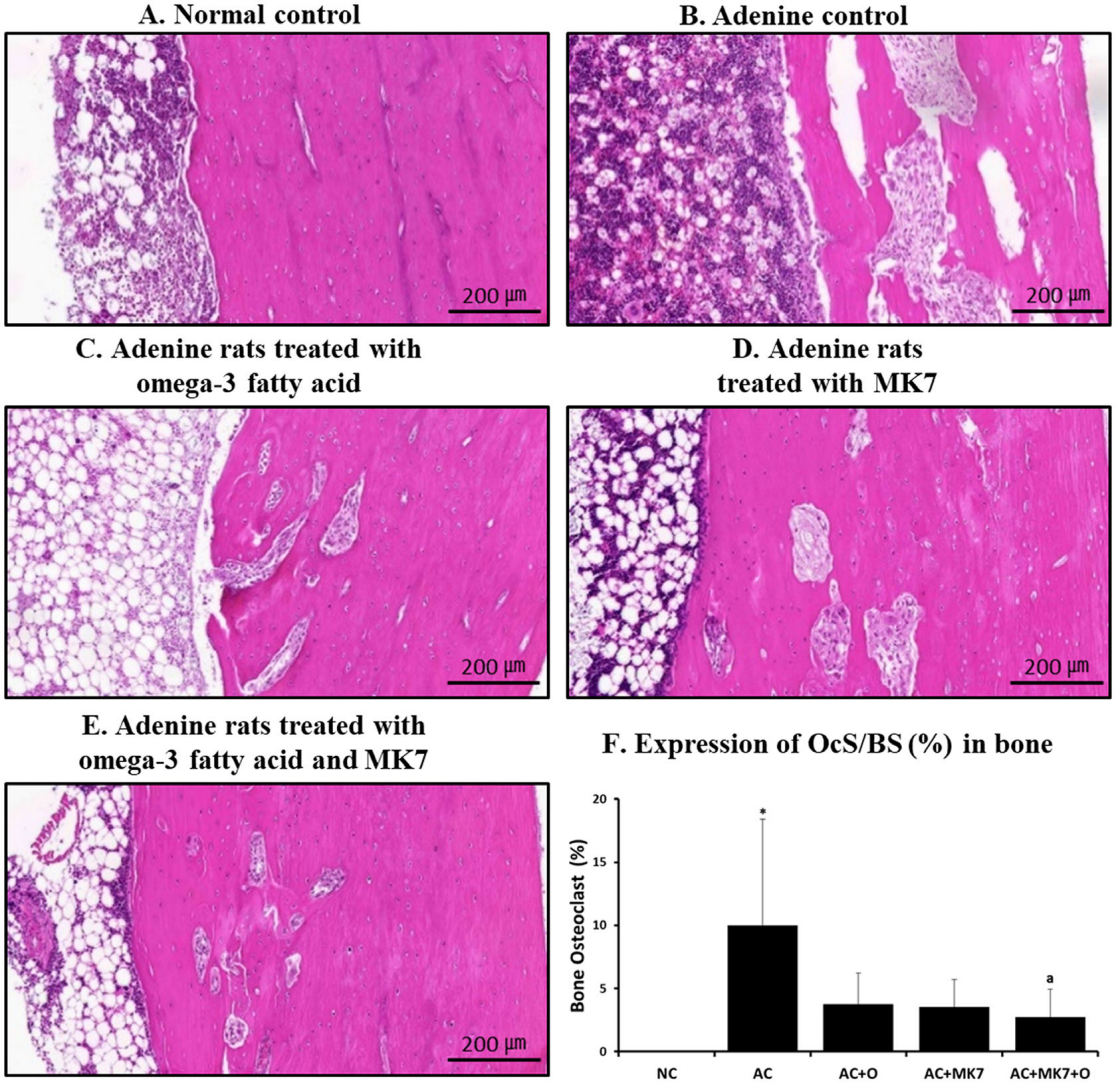
Osteoclast surface/bone surface ratio using H&E stain. Osteoclast surface/bone surface ratio (OcS/BS %) was significantly higher in adenine control group compared to normal control and significantly lower in combination supplementation group compared to adenine control. *p < 0.05 (mean values are significantly different from normal control). ^a^*p* < 0.05 (mean values are significantly different from adenine control).

The OcS/BS was significantly higher in the adenine control group than in the normal control group (Figure 3). The OcS/BS was significantly lower in the combined omega-3 FA and MK-7 supplementation group than in the adenine control group.

### Expression of factors related to sarcopenia

**Figure 4.**
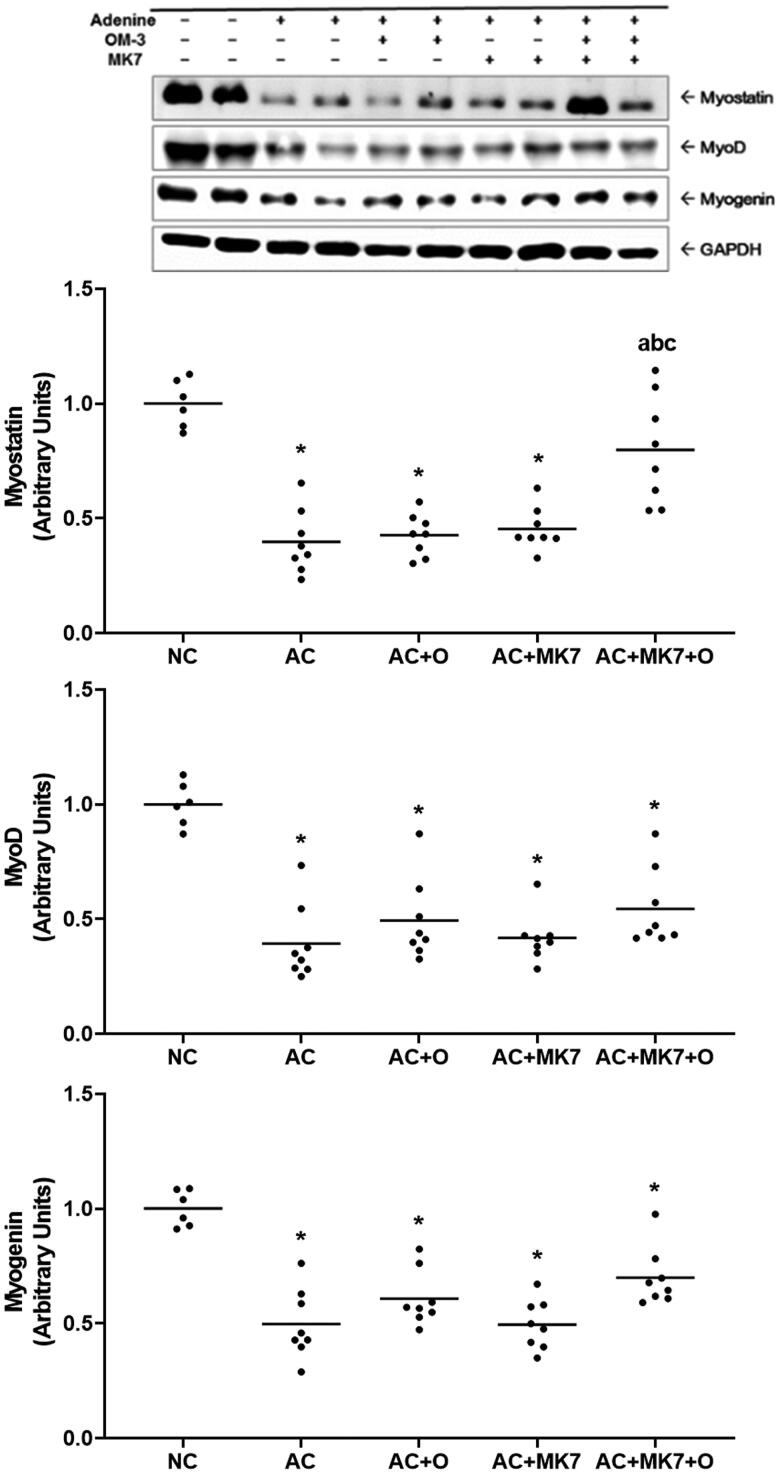
Expression of myostatin, myoD and myogenin on Western blot analysis. Myostatin in adenine control group was significantly restored by combined supplementation with omega-3 FA and MK-7. *p < 0.05 (mean values are significantly different from normal control). ^a^*p* < 0.05 (mean values are significantly different from adenine control). ^b^*p* < 0.05 (mean values are significantly different from adenine rats supplemented with omega-3 FA). ^c^*p <* 0.05 (mean values are significantly different from adenine rats supplemented with MK-7).

Compared with normal control group, the adenine control group presented significantly down-regulated myostatin, myoD, and myogenin in the western blot analysis of muscle. The decreased myostatin expression in the adenine control group was significantly restored in combined supplementation group with omega-3 FA and MK-7. However, myoD and myogenin were not significantly recovered by the combined supplementation (Figure 4).

**Figure 5.**
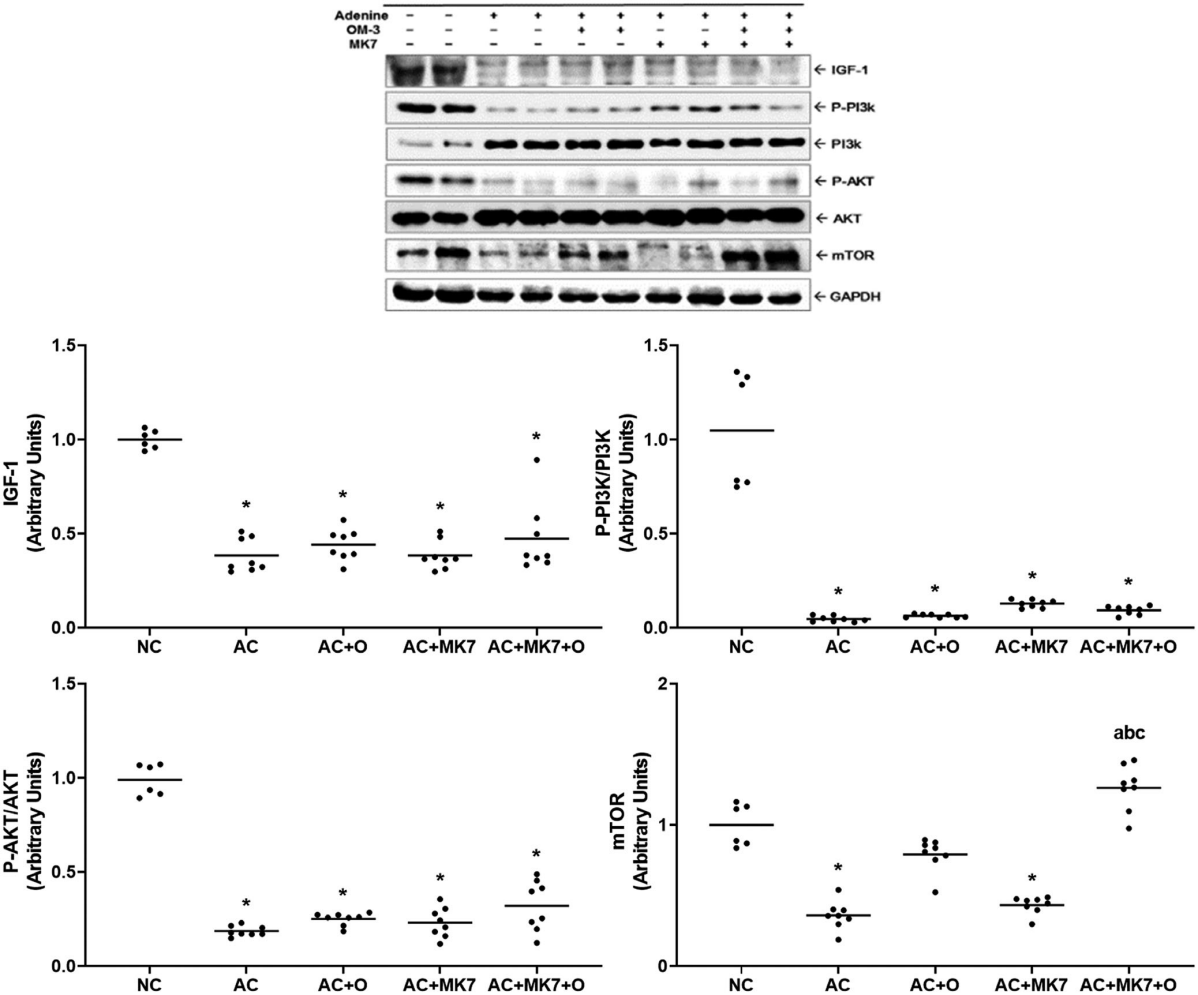
Expression of IGF-1, p-PI3K, p-Akt, mTOR on Western blot analysis. IGF-1, p-PI3K, p-Akt and mTOR were significantly down-regulated in the adenine control group. Decreased expression of mTOR were restored by combination supplementation with omega-3 FA and MK-7. *p < 0.05 (mean values are significantly different from normal control). ^a^*p* < 0.05 (mean values are significantly different from adenine control). ^b^*p* < 0.05 (mean values are significantly different from adenine rats supplemented with omega-3 FA). ^c^*p* < 0.05 (mean values are significantly different from adenine rats supplemented with MK-7).

IGF-1 and phosphorylated Akt were downregulated in the adenine control group, compared to the normal control group; however, no significant changes were found in the supplementation groups. Compared to the normal control group, the adenine control group presented significantly downregulated mTOR expression. The decreased mTOR expression was restored by omega-3 FA, but not by the MK-7 individual supplementation. Combination supplementation group with omega-3 FA and MK-7 showed synergistic effects in increasing mTOR expression (Figure 5).

**Figure 6.**
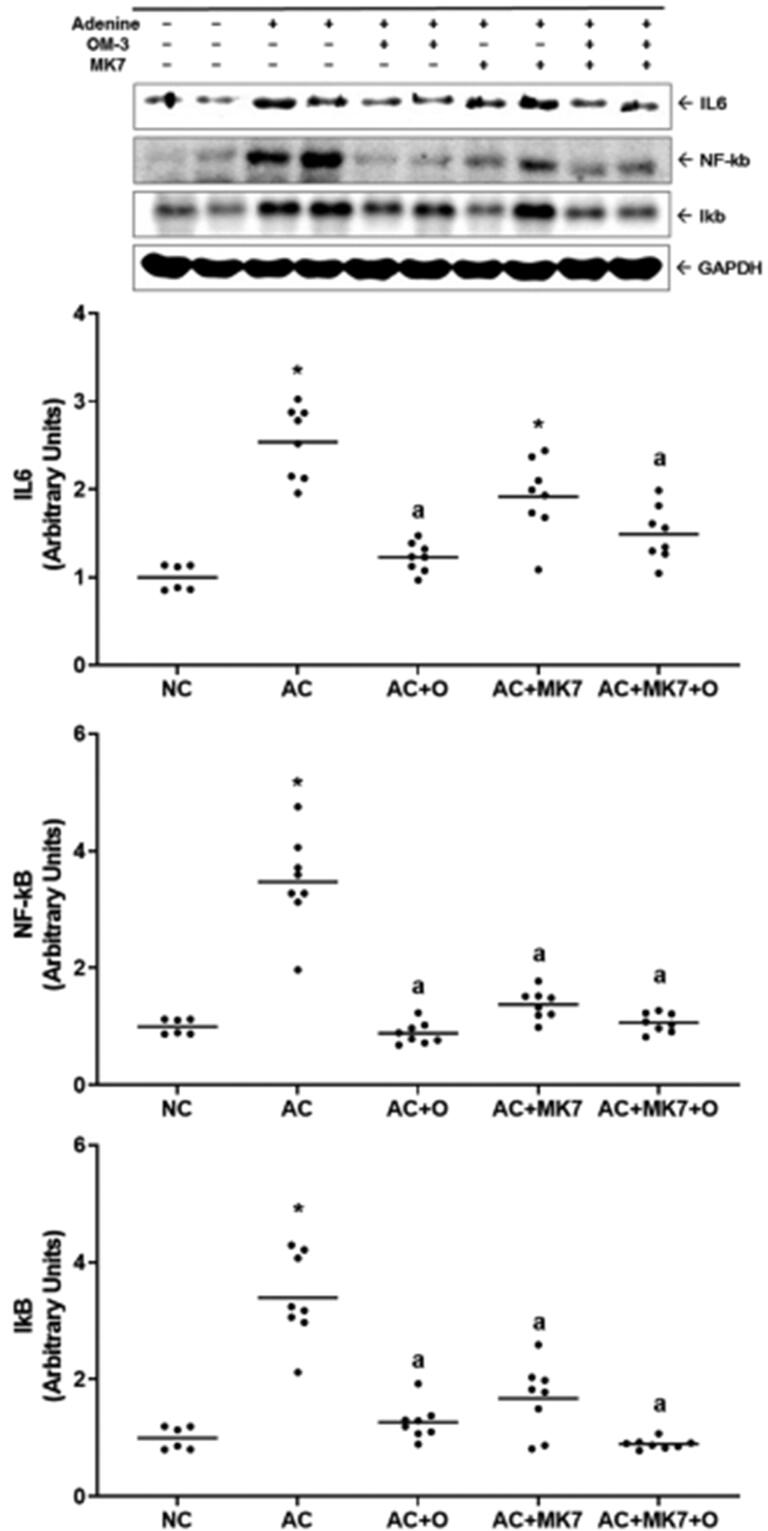
Expression of IL6, NF-kB, IkB on Western blot analysis. IL-6, NF-kB, and IkB were up-regulated in the adenine control. IL-6 was dominantly counteracted by supplementation with omega-3 FA. NF-kB and IkB were up-regulated in the adenine control and they mostly counteracted by combination supplementation with omega-3 FA and MK-7. *p < 0.05 versus normal control. ^a^*p* < 0.05 versus adenine control.

Inflammatory cytokines, such as IL-6, NF-kB, and IkB, were upregulated in the adenine control group compared to the normal control group (Figure 6). Changes in IL-6 levels were restored after supplementation with omega-3 FA or omega-3 FA and MK-7; however, they were unaffected by MK-7. In contrast, NF-κB and IkB expression was restored after supplementation with omega-3 FA, MK-7, or omega-3 FA and MK-7.

**Figure 7.**
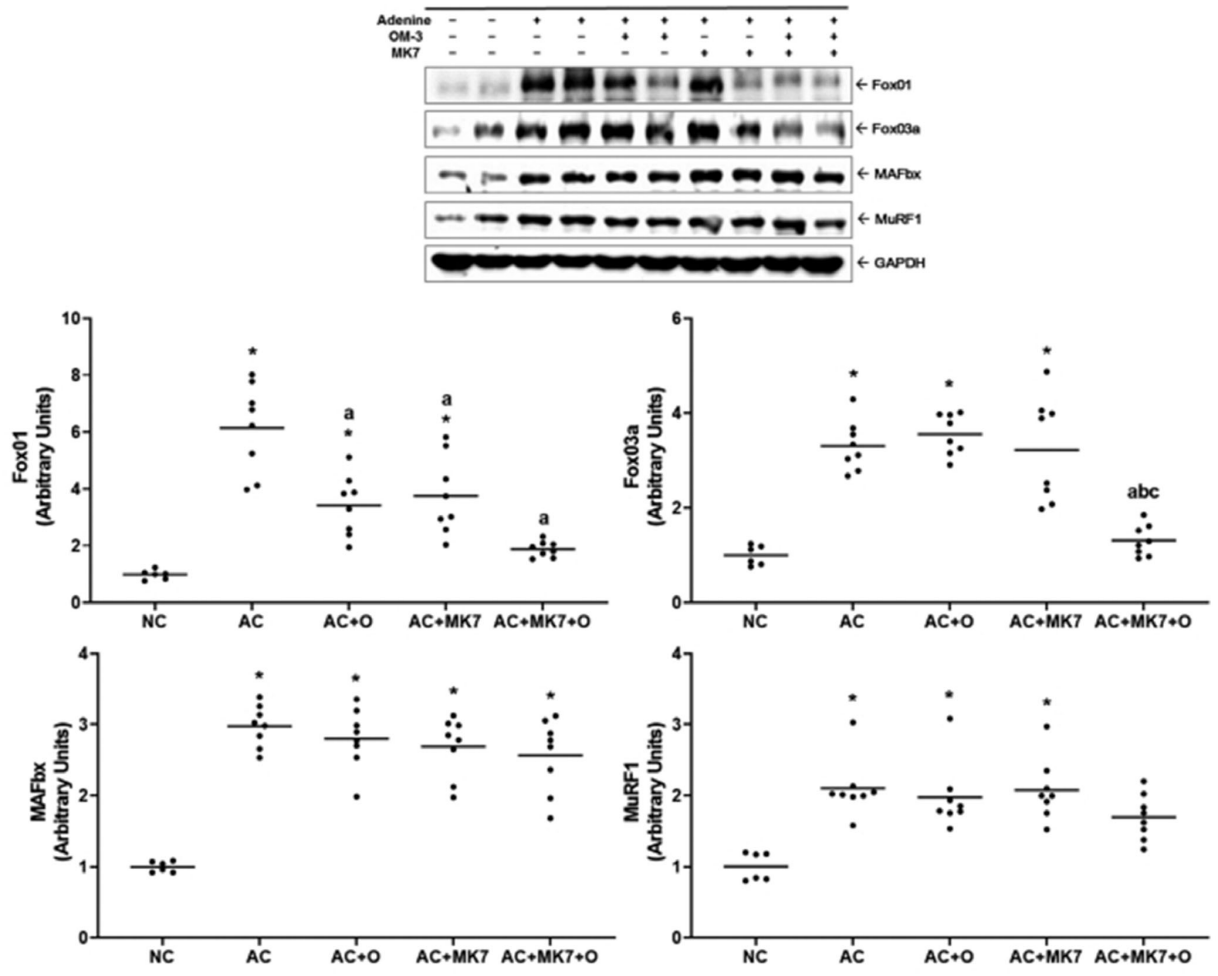
Expression of Fox01, Fox03a, MAFbx, MuRF1 on Western blot analysis. Fox01 and Fox03 were up-regulated in the adenine control group. Fox01 and Fox03a were down-regulated by combination supplementation with omega-3 FA and MK-7. *p < 0.05 (mean values are significantly different from normal control). ^a^*p* < 0.05 (mean values are significantly different from adenine control). ^b^*p* < 0.05 (mean values are significantly different from adenine rats supplemented with omega-3 FA). ^c^*p* < 0.05 (mean values are significantly different from adenine rats supplemented with MK-7).

Fox01 and Fox03a, which activate protein degradation, were upregulated in the adenine control group (Figure 7). Fox01 and Fox03a were downregulated by the omega-3 FA and MK-7 combination supplementation. MAFbx and MuRF1 were upregulated in the adenine control group, but no significant changes were found after supplementation with omega-3 FA or MK-7

### Pathology of muscle

**Figure 8.**
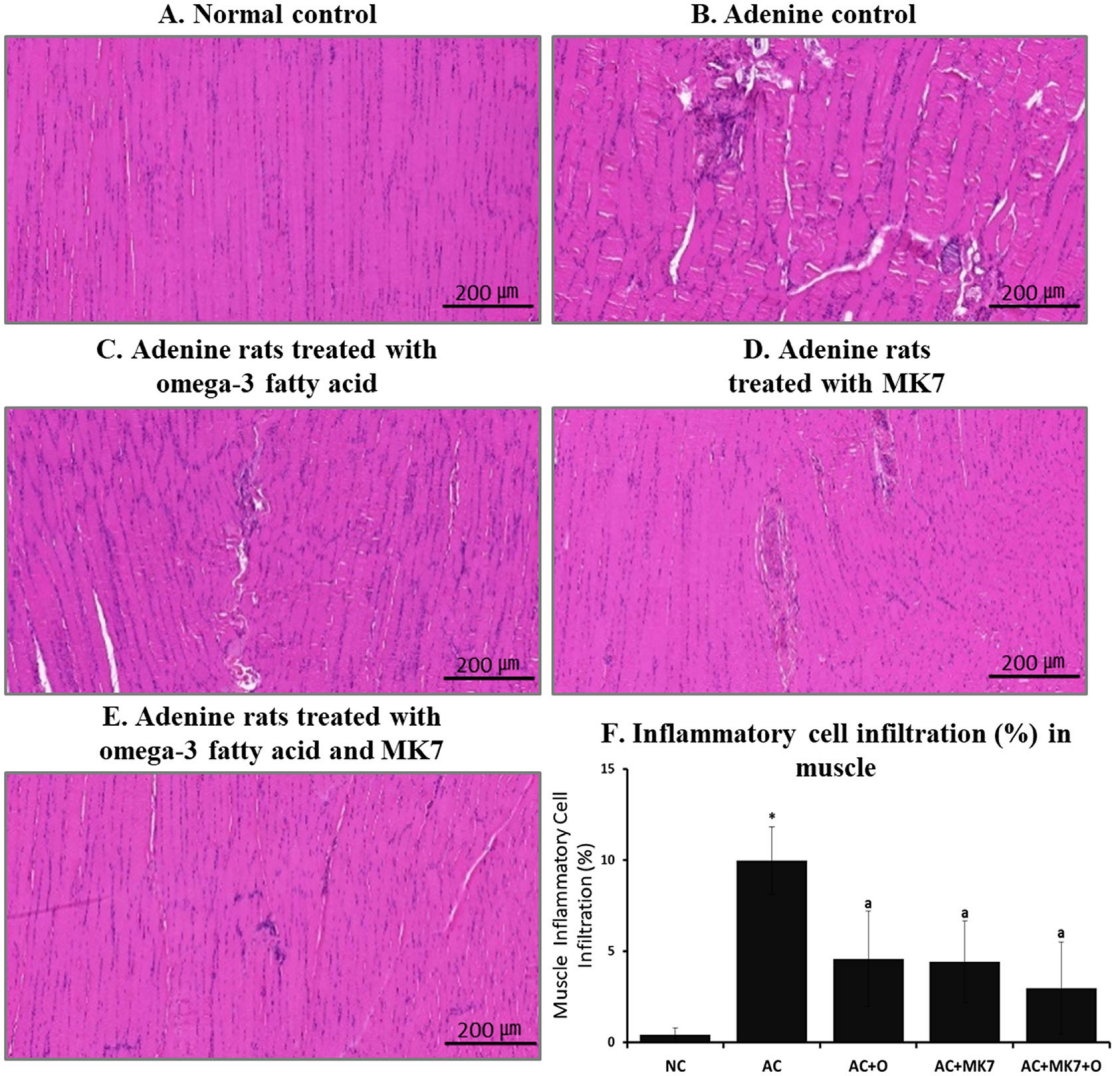
Histologic findings of calf muscle using H&E stain. The regular arrangement of muscle fiber was destroyed and inflammatory cell infiltration was more prominent in adenine control group compared to normal control. These findings were partially diminished after combination supplementation with omega-3 FA and MK-7.

From the calf muscle histology findings using H&E staining, muscle fiber thickness did not significantly differ between the adenine control and supplementation groups. However, the regular arrangement of muscle fiber was severely altered in the adenine control group, compared to the normal control group (Figure 8). Inflammatory cell infiltration was also more prominent in the adenine control group than in the normal control group. These findings were diminished after combined supplementation with omega-3 FA and MK-7.

## Discussion

In this study, less progressed aortic calcification and lower osteoclast area of bone were found in a severe uremic rat model supplemented with both omega-3 FA and MK-7. We used a rat model fed with 0.75% adenine and 2.5% low-protein diet to induce CKD-related bone status and VC. A previous study showed that VC was prevented by bisphosphonate due to inhibition of bone resorption in a low-protein and adenine-induced CKD model [15]. Low-protein diet has been demonstrated to increase VC in uremic rats and to increase bone resorption leading to bone loss [16,17]. Based on this study, combined omega-3 FA and MK-7 supplementation may be beneficial for preventing bone loss and VC progression in advanced CKD patients usually recommending low-protein or very low-protein diet in the clinical setting. Vitamin K is a cofactor for activation of MGP and MGP requires carboxylation, which is completely dependent on vitamin K [18]. In cases of vitamin K deficiency, MGP is not activated and uncarboxylated MGP is usually accumulated. These conditions are related to intimal and medial calcification [19]. Previous clinical studies have also shown that menaquinone supplementation was related to decreased coronary artery calcification (CAC) and decreased serum uncarboxylated MGP concentrations [20,21]. Considering the cross-talking between VC and bone resorption, vitamin K is also expected to have a positive effect on the prevention of osteopenia as well as VC. MK-7 single supplementation also reduced aortic calcification, compared to the adenine control group, and reduced osteoclast surface in this study. Omega-3 FA attenuates arterial medial calcification by inhibiting macrophage infiltration and osteogenic protein expression in warfarin-induced rat models [22]. Also, omega-3 FA can reduce osteoblastic differentiation through the activation of the peroxisome proliferator-activated receptor-γ [23,24]. Fetuin-A, reported as a circulating inhibitor of VC, was increased by omega-3 FA supplementation in dialysis patients [25]. Similar to previous study, omega-3 FA single supplementation also reduced aortic calcification and additionally reduced osteoclast surface in this study. It is of note that the synergistic effect of MK-7 and omega-3 FA on decreasing osteoclast surface and reducing aortic calcification was demonstrated in this study.

OPG is a decoy receptor for the receptor activator of NF-kB ligand and is expressed in osteoblast lineage cells. OPG can interfere with the production of osteoclasts by inhibiting the process of osteoclast precursors differentiating into mature osteoclasts. They also regulate the resorption by osteoclasts [26]. Both bone density reduction and VC was shown in OPG-deficient mice [27]. In experimental animals, over-expression of the *OPG* gene prevents VC and OPG and functions as a protective factor for VC. However, in human epidemiological studies, increased serum OPG levels and uremia correlate with increased CAC [28]. OPG levels positively associate with CAC and lead to CVD in patients on dialysis as well as the general population [29]. Activated osteoclasts and vascular injury may promote OPG secretion by osteoblasts as a reactive or compensatory mechanism. In addition, a higher OPG level may reflect a higher proportion of osteoblast-like vascular cell functions, the secretion of OPG, and the active status of osteoclasts [30,31]. In this study, a higher OcS/BS with strong OPG staining may reflect activated osteoclasts on bone area. Higher aorta VC area with strong OPG staining may be explained by higher proportion of osteoblast like cells in the aorta of adenine control group. We suspect that less OcS/BS area and less OPG stain of bone reflect less activation of osteolclast in the combined supplementation with omega-3 and MK-7 groups. In addition, less aortic VC area and less OPG stain indicate less proportion of osteoblast-like vascular cell transition in the combined supplementation with omega-3 and MK-7 groups.

Sarcopenia is commonly observed in patients on dialysis and highly correlated with morbidity and mortality [32]. Skeletal muscle and bone loss are interrelated by both biomechanics and common exposure to uremic toxins. Myostatin, a member of the transforming growth factor beta superfamily, is a protein produced and released by myocytes that acts on muscle cell autocrine function to inhibit muscle cell growth and differentiation [33]. Myostatin is a negative regulator of skeletal muscle mass, and an imbalance between IGF-1 and myostatin has been observed in experimental uremia models [34]. Myostatin expression is increased in several diseases that demonstrate skeletal muscle wasting, including CKD [35]. However, serum myostatin values are negatively correlated with abdominal aortic calcification of an aged man [36]. A recent study showed that lower serum myostatin levels were correlated with higher VC scores, lower muscle mass, and lower bone density in patients on dialysis [37]. Similar findings were observed in this animal study. Compared with normal control group, the decreased myostatin expression, increased aortic calcification and increased OcS/BS area were found in the adenine control group. Myostatin is expressed in the heart and adipose tissue but is mainly synthesized and excreted by skeletal muscles [38]. Therefore, inflammatory changes and muscle fiber destruction, which reflect muscle damage, could result in a low production and secretion of myostatin from myocytes. It could be assumed that lowered myostatin expression may be a manifestation of severely decreased myocyte caused by severe uremic and inflammatory status in adenine and low-protein diet induced uremic rats. Further studies are needed to elucidate the mechanisms and its potential clinical implications.

Muscle wasting is caused by an increase in inflammation and reactive oxygen species, which elevate the signaling of protein degradation through several important pathways. FoxO transcription factors, which activate the ubiquitin-proteasome system (UPS) and autophagy, is one of these pathway [39]. Furthermore, sarcopenia is correlated with lower levels of IGF-1 and lowered IGF-1 damages protein synthesis by inhibiting the phosphatidylinositol 3-kinase (PI3K)-Akt-mTOR pathway [40]. Despite decreased IGF-1 expression and no restored Akt phosphorylation, inflammatory cytokines were recovered by omega-3 FA or MK-7 supplementation in this study. Therefore, it is assumed that both omega-3 FA and MK-7 combination supplementation improve FoxO and mTOR by dominant anti-inflammatory effect. UPS is a major skeletal muscle pathway for protein degradation. The E3-ubiquitin ligases, MuRF-1 and MAFbx, represent two of the principal ligases in skeletal muscle that check proteins for elimination through the UPS [41]. The increased activity of the proteasome pathway in sarcopenia seems to be mediated by activation of the FoxO and NF-ĸB transcription factors, which lead to MuRF1 and MAFbx expression and induce increased proteasome activity [39]. In this study, Fox01 and Fox03a, which activate protein degradation, were upregulated in the adenine control group, and they were restored by combination supplementation with omega-3 FA and MK-7. However, the expression of MAFbx and MuRF1, the final regulators of protein degradation, were not affected by any supplementation (neither omega-3 FA nor MK-7). Therefore, there was no improvement in muscle fiber thickness in the histology findings of omega-3 FA and MK-7 supplementation group. Further studies are needed to clarify this phenomenon especially in less uremic animal model.

Conclusively, combined nutrients supplementation with omega-3 FA and MK-7 may be helpful for aortic VC prevention, reducing osteoclast activation and improving inflammation, and sarcopenia-related molecules in adenine and low-protein diet induced uremic rats.

## Data Availability

The datasets used and analyzed during the current study are available from the corresponding author on reasonable request.
